# A Review of the Role of Curcumin in Metal Induced Toxicity

**DOI:** 10.3390/antiox12020243

**Published:** 2023-01-21

**Authors:** Elena Smirnova, Mohammad Moniruzzaman, Sungyeon Chin, Anjana Sureshbabu, Adhimoolam Karthikeyan, Kyoungtag Do, Taesun Min

**Affiliations:** 1 Department of Animal Biotechnology, Jeju International Animal Research Center (JIA) & Sustainable Agriculture Research Institute (SARI), Jeju National University, Jeju 63243, Republic of Korea; 2 Subtropical Horticulture Research Institute, Jeju National University, Jeju 63243, Republic of Korea

**Keywords:** curcumin, trace elements, antioxidant defense, chelation therapy, reactive oxygen species, hepatoprotective effect, oxidative stress, neuroprotective effect

## Abstract

Metal toxicity poses a potential global threat to the environment and living beings. Their numerous agricultural, medical, industrial, domestic, and technological applications result in widespread distribution in the environment which raises concern on the potential effects of metals in terms of health hazards and environmental pollution. Chelation therapy has been the preferred medical treatment for metal poisoning. The chelating agent bounds metal ions to form complex cyclic structures known as ‘chelates’ to intensify their excretion from the body. The main disadvantage of synthetic chelators is that the chelation process removes vital nutrients along with toxic metals. Natural compounds are widely available, economical, and have minimal adverse effects compared to classical chelators. Herbal preparations can bind to the metal, reduce its absorption in the intestines, and facilitate excretion from the body. Curcumin, a bioactive substance in turmeric, is widely used as a dietary supplement. Most studies have shown that curcumin protects against metal-induced lipid peroxidation and mitigates adverse effects on the antioxidant system. This review article provides an analysis to show that curcumin imparts promising metal toxicity-ameliorative effects that are related to its intrinsic antioxidant activity.

## 1. Introduction

Metals contamination has become an urgent threat due to its persistence, cumulative effect, and specific toxic properties [1]. Most of the metals released into the environment are of anthropogenic origin, but they are also detected in natural sources such as water, soil, and rocks. They are mostly considered as conservative pollutants because they are chemically inert and have no detrimental impact unless disturbed [2]. However, the intensive commercial and industrial development has led to the significant changes in the biogeochemical cycles of metals, which gave rise to the most powerful sources of pollution in the biosphere [3].

**Figure 1:**
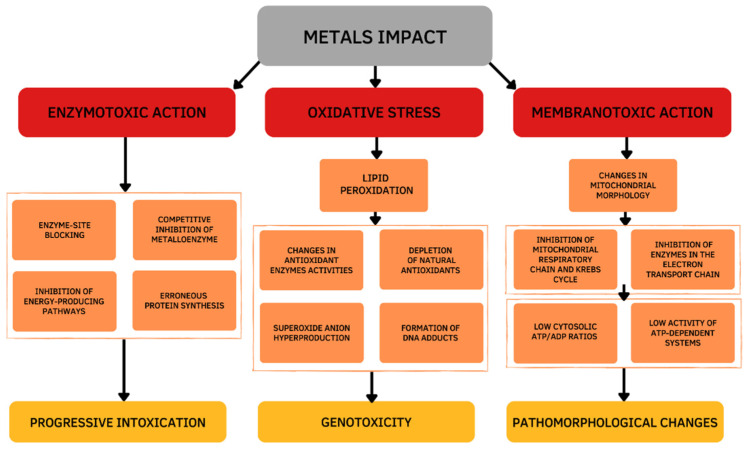
Cellular mechanisms of the metals toxic action.

Damage effects and mechanisms of various metals on the human body are similar. Excessive exposure to metals is dangerous and has many several acute and chronic toxic effects on different organs, particularly on the liver. The main changes occur in the oxidative and antioxidant systems under the influence of the metal toxicity are based on three mechanisms (Figure 1). The first mechanism is determined by the ability of metals to bind functional groups of biologically active substances in the body, primarily blocking the sulfhydryl (SH) groups of Cys-containing enzymes. As a result of the reaction of metal ions with SH-groups, weakly dissociating and insoluble compounds are formed such as mercaptides (a metallic salt of methanethiol). The formation of mercaptides is accompanied by damage and dysfunction of proteins, which initiates the development of a toxic process [4]. The second mechanism of the toxic action is based on the displacement of biogenic metals from metal-containing complexes. If the stability of the metal complex is greater than the stability of the biogenic metal complex, the metal is transferred to the more stable complex and metal compounds accumulate in the body, which leads to the disruption of the normal functioning of the body. This mechanism is due to the larger binding strength of toxic vs. biogenic metal ions [5]. The third mechanism is determined by the development of oxidative stress. Active oxidizing-reducing metals (e.g., Fe, Cu, Cr, Co) are directly involved in redox reactions in cells, resulting in the formation of superoxide ions (O_2_^•−^). Then, hydrogen peroxide (H_2_O_2_) and hydroxyl radical (•OH) are formed during the Fenton reactions and the Haber–Weiss cycle. Exposure to redox inactive metals (e.g., Cd, Zn, Ni, Al) leads to the oxidative stress through the interaction with the antioxidant defense system, disruption of the electron transport chain, or induction of lipid peroxidation [6].

Thus, the toxicity of metals is based on enzymatic toxicity due to the inhibitory action of metalloenzymes, membranotropic action, and oxidative stress. The chemical basis of the metal’s toxicity is its ability to bind functional groups of biologically important substances of the body, displace essential metals from metal-containing complexes, and generate reactive oxygen species (ROS). Mechanisms of toxicity are not mutually exclusive and may occur simultaneously.

Due to their bioaccumulation ability, metals pose a severe threat to the body if they exceed acceptable limits (Table 1). Since they are not biodegradable, this only increases affecting health by remaining in the body for a longer time and presenting a long-term risk [3]. To combat organ toxicity caused by metals, scientists are looking for suitable protective agents. The most common chelators are ethylenediaminetetraacetic acid (EDTA), succimer (2,3-dimercaptosuccinic acid, DMSA), unithiol (DMPS), N-acetylcysteine (NAC), alpha-lipoic acid (ALA), etc. [7,8]. In recent years, trends in using nutritional antioxidants have attracted increasing interest. Plant products are well known to protect cells from the aggressive action of free radicals and strengthen the antioxidant defense systems [9]. Certain phytochemicals—including flavonoids and tannins—have metal binding activity for cadmium, nickel, aluminum, and lead in vitro and in vivo. Apart from these, other antioxidants—such as vitamin C, vitamin E, beta-carotene, etc.—may also be useful in combating metal toxicity in animals [10,11]. The beneficial influence of curcumin, a natural bioactive and antioxidant-extracted turmeric spice, on prevention and recovery from metal intoxication, remains to be defined.

This review analyzes the research concerning the valuable and controversial features of curcumin to determine the effectiveness of its implementation in the therapeutic treatment of metal toxicity and aims to bring researchers’ attention to the most noticeable data obtained in recent years.

## 2. Curcumin as a Bioactive Compound in Turmeric Plant

Curcumin (CUR) is a hydrophobic low molecular polyphenolic compound [25], a bright yellow color pigment extracted from the rhizome of turmeric (family: Zingiberaceae). It is often considered the most active natural component among other compounds in the plant, comprising about 2–8% of most turmeric formulations [26]. One of the most frequently chosen as an object of study among natural products in different countries [9,27]. Due to its therapeutic and prophylactic properties, it has gained popularity as part of traditional Indian and Chinese medicine [28]. Various studies have presented that the therapeutic potential of CUR does not cause adverse effects in animal models or humans [29]. Clinical and laboratory tests have shown that CUR has a favorable safety profile and can be well tolerated. Treatment even at high doses did not cause negative consequences as well as non-toxic [30,31].

**Figure 2:**
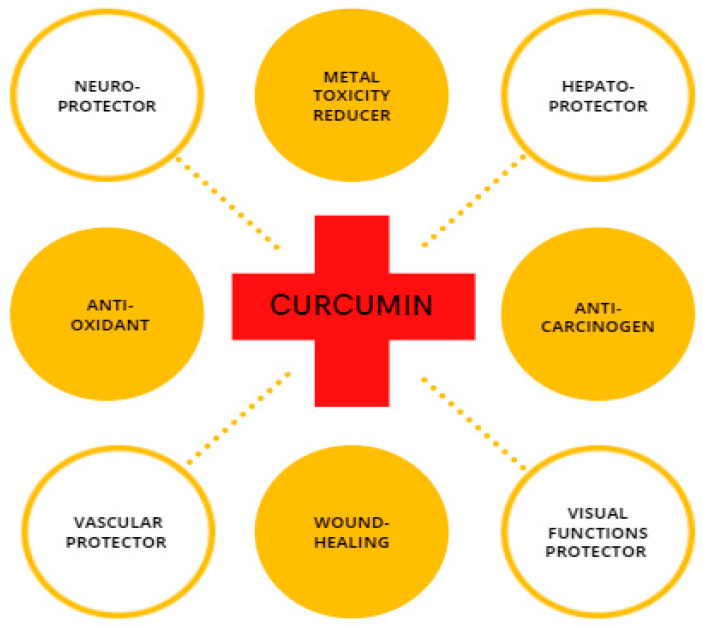
Schematic illustration of CUR with its biological properties.

CUR is a compound with high physiological activity, which is intensively studied as a protective element in the field of cancer research [32,33,34]; steatohepatitis [35]; atherosclerosis [36]; neurodegenerative disorders, such as Parkinson’s disease [37,38]; and Alzheimer’s disease [39,40] along with for repairing damaged skin [41,42,43] (Figure 2). Recently, researchers have considered CUR as a neuroprotector in cerebral ischemia-reperfusion. CUR protects the mitochondria of nerve cells from the damaging effects of β-amyloid [44]. Experimental animal studies have shown that turmeric consumption inhibits the process of memory deterioration during brain aging [45]. It also protects the hippocampus from the damaging effects of the long-term use of dexamethasone [46]. Considering the protection effect of CUR against memory impairment [47], it can prevent the development of oxidative stress in the nervous tissue under the influence of sodium nitroprusside [48]. In experimental animals, CUR reduced movement disorders and stiffness caused by haloperidol [49]. Experimental studies have shown that turmeric extract prevents addiction during long-term use of morphine effect in rats [50]. Many studies have demonstrated the antioxidant and anti-apoptotic effects of CUR [51,52]. The involvement of CUR in biological processes mostly arises from its ability to either modulate the intracellular redox state or straight interact with proteins, such as cyclooxygenase 2 (COX-2) [53]. CUR provides forceful antioxidant protection by strongly suppressing the formation of ROS, thereby the organism can counteract oxidative tissue damage [54]. Experiments using animal models for diabetes indicate that the CUR supplement improves microcirculation and decreases blood lipid and glucose levels [55]. In addition to the pharmacological effects of CUR, it has a protective effect against oxidative damage caused by metal toxicity in the reproductive organs [56,57].

## 3. Chemical Properties of Curcumin

**Figure 3:**
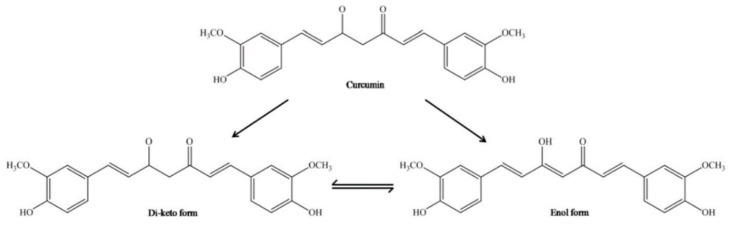
Chemical structure of enol and keto tautomeric forms of CUR molecule [61].

CUR has an original chemical structure that allows it to participate in a range of physical, chemical, and biological processes. Chemically, CUR or diferuloylmethane (1,7-bis-(4-hydroxy-3-methoxyphenyl)-1,6-heptadiene-3,5-dione) is a bis-α, β-unsaturated β-diketone, resulting from the conjugation of two ferulic acid molecules connected via a methylene bridge. There are three main functional groups: an aromatic ring, β-diketone, and an alkene bridge. The β-diketone provides flexibility, and the aromatic rings provide hydrophobicity. Conducted research using a molecular modeling technique has shown that CUR may advantageously transform to maximize hydrophobic contact with an interacting protein. The CUR molecule is a diketone that exhibits keto-enol tautomerism. In nonpolar solutions and in a solid phase, CUR exists in the enol form stabilized by hydrogen bonds [58,59]. The interconversion between tautomeric forms of CUR gives supplementary chemical functionality (Figure 3). The most reactive enol form can act both as a donor and an acceptor in the formation of hydrogen bonds. In addition, the keto-enol site determines the advantage of CUR’s chelating activity of divalent ions [60]. It should be noted here that only deprotonated or enolate form of the enol isomer has metal chelating ability through the exhibition of the metal binding properties. 

## 4. Bioavailability of Curcumin

Despite the powerful pharmacological effects of CUR, it is necessary to point out that its biomedical potential cannot be fully revealed owing to its low bioavailability. The main disadvantages of CUR are the poor oral bioavailability in terms of low intestinal absorption rate, rapid metabolism, and excretion from the body [62] due to the hydrophobic (low solubility in water) and lipophilic (highly soluble in lipid) nature of CUR [63]. It has been reported that about 75% of dietary CUR can be excreted through the feces of animals [64]. 

**Figure 4:**
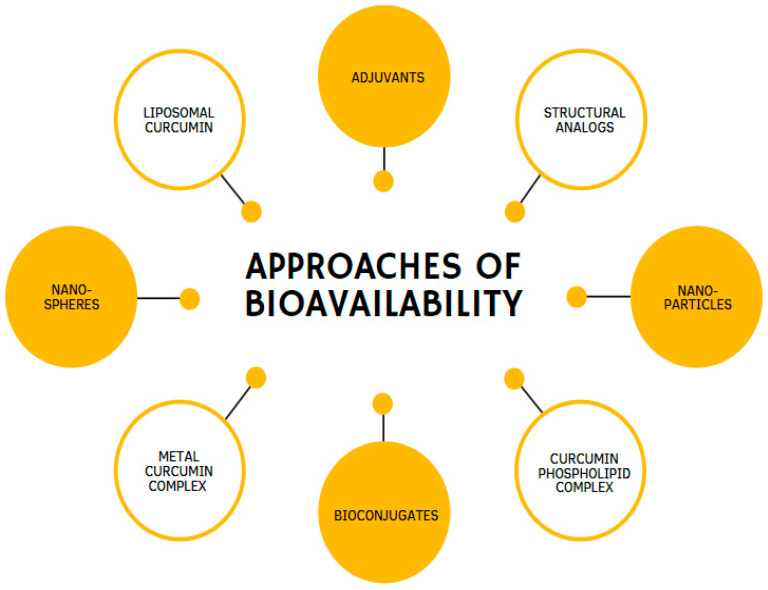
Different methodologies are used to enhance the bioavailability of curcumin.

Over the past few years, a considerable number of approaches have been created to improve CUR’s potency and effectiveness. These approaches propose various methods: the use of enhancers, compounds that promote the delivery of biologically active substances, such as the alkaloid piperine [65]; CUR incorporation into liposomes [66]; phospholipids loaded with CUR [67]; the use of analogs that have structural and pharmacological similarities of CUR [68]; and CUR incorporation into nanoparticles [69,70] (Figure 4). Moreover, remarkable results have been achieved by combining CUR in complexes with cyclodextrins [71] or phosphatidylcholine [10,72]. 

Integrating CUR into various nanosystems is the most promising and productive technique to improve the biological activity of poorly water-soluble CUR. With the help of such systems, the highest plasma concentrations, a stable release profile, and significantly increased relative bioavailability have been achieved [73,74,75,76]. The results showed the effectiveness of the obtained nanoparticles compared with pure CUR [77]. It has been reported that PLGA-nanocurcumin is superior to poorly bioavailable native curcumin at 15-fold lower concentration in therapeutic application [78]. Moreover, Shaikh et al. [79] found that encapsulation of nanocurcumin is 9-fold more suitable than native curcumin in terms of oral bioavailability in animals.

A particularly innovative method to address the problem of poor oral absorption is metal complexes with CUR. The enolate form of CUR has ability to bind metal ions enriches the CUR molecule with a chelating mechanism that contributes to the antioxidant activity [80].

## 5. General Perspective of Curcumin in the Protection of Metal Toxicity

**Figure 5:**
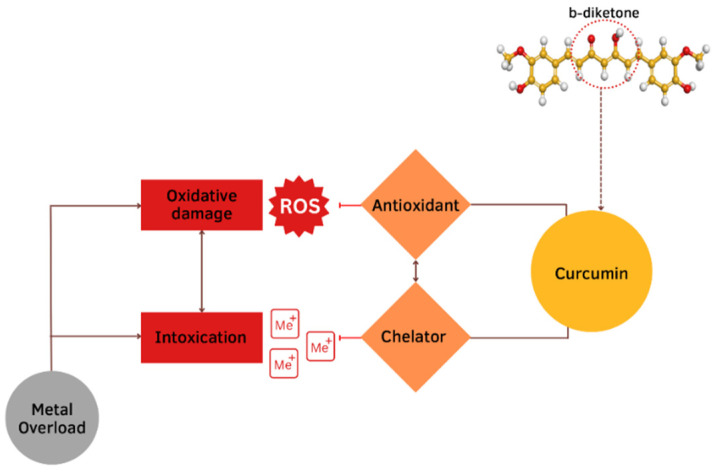
Mechanisms of the protective effects of CUR against metal toxicity. The protective effect of CUR is ascribed to its neutralization of free radicals and chelating properties. This is achieved primarily due to the presence of a b-diketone.

**Figure 6:**
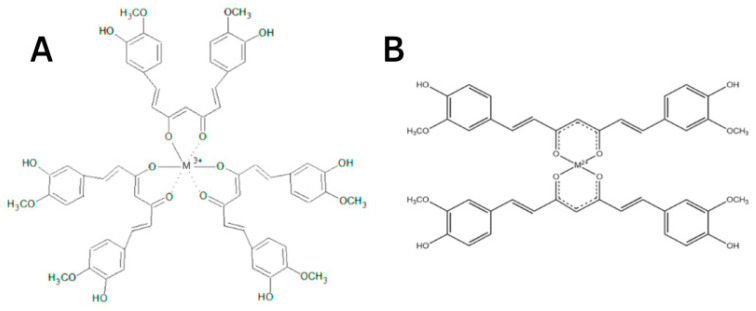
A simplified illustration of the CUR chelating trivalent metals in a 1:3 configuration (**A**) and bivalent metals in a 1:2 configuration (**B**) (adapted from Rainey et al. [94]).

The protective effect of CUR is ascribed to its neutralization of free radicals and chelating properties [81]. CUR can act at the same time as a metal chelator and antioxidant. The CUR chemical structure allows to form chelate complexes with high stability, thereby removing toxic metal ions and inhibiting Aβ polymerization and following the generation of toxic conformations [82]. This is achieved primarily due to the optimal properties of CUR’s natural complexing agent and the presence of a 1,3-diketone moiety that provides the tautomeric form. The more stable enol form acts as a complexing agent [83] with high binding activity and coordinates metal ions around itself [84,85,86], creating complexes [87,88] (Figure 5). The presence of this form makes it an appropriate chelating agent for ingested toxic metals. The keto-enol part is the reaction center [84,85,86,87,88] and provides protection against active free radicals [88]. The enol form of the diketone is better stabilized due to charge delocalization and generates 1:2 and 1:3 type chelates with metal ions [89,90,91,92] (Figure 6). CUR, owing to its clear lipophilicity, easily passes through the blood–brain barrier and cell membranes, which makes it possible to scavenge toxic metals intracellularly [93].

## 6. Curcumin on Aluminum-Induced Toxicity

Aluminum is ubiquitous in the environment in the form of salts and oxides. Al can enter the organism with drinking water, air, and plant food. One of the Al-specific sources is its ever-increasing use in the food industry (e.g., dishes, packaging material, food additives) and pharmacology [95]. At the molecular level, it can cause protein precipitation and the formation of insoluble protein compounds, which affects reducing the enzyme activity and their systems [96]. Moreover, Al intoxication leads to changes in blood composition, blood disorders (lymphocytosis, eosinopenia, anemia), disorders of calcium-phosphorus metabolism, decreased stability of DNA synthesis, DNA damage, and development of soft tissue fibrosis [97,98]. Furthermore, Al is known as a neurotoxic metal, which may show its negative effects on the nervous system, particularly at higher concentrations; causing movement disorders, seizures, memory loss, psychopathic traits, learning disabilities, depressive tendencies, and encephalopathy [99]. Al is assumed to play a significant role in the occurrence of severe neurodegeneration such as Alzheimer’s disease [95,100]. One of the treatment approaches for Al-induced neurotoxicity is the search for antagonists that halt the absorption of metal [101], replenish nutritional deficiency, and prevent the launching of oxidative processes caused by ROS exposure and disruption or depletion of the antioxidant system during Al intoxication. 

It was found that CUR interacts strongly with Al (III), implying that CUR has the potential of removing Al (III) ions and averting interaction between Al (III) and amyloid β-protein (Aβ); thereby, stopping the toxicity of Aβ and detrimental effects of oxidative stress [102] (Table 2). CUR reduced the bonding affinity of Al^3+^ to DNA [103]. Memory improvement, reversing oxidative damage, and weakening of acetylcholinesterase activity have been observed with CUR long-term administration. Moreover, CUR prevented damage to neurons under oxidative stress conditions, induction of Al^3+^-caused cognitive impairment, and markedly lowered the Al concentration in Al-treated rats [104]. CUR administration led to a decrease in the expression of NF-κB and TNF-α which are known as the markers of inflammatory reactions [100]. Furthermore, curcumin treatment led to the reduction in ROS and lipid peroxidation in cerebellum as well as improvement of reduced glutathione and glutathione-S-transferase in cerebrum which showed the neuroprotective behavior of curcumin on Al-induced neurodegenerative and behavioral disorders in rats [105]. Recently, it is reported that nano-curcumin has better biological and antioxidant activity than native curcumin on Al-induced toxicity in rats [106] which might be due to the favorable interaction of nanoparticle of curcumin with Al^3+^ in reducing Al toxicity. 

## 7. Curcumin on Arsenic-Induced Toxicity

Arsenic is a commonly occurring metal in the biosphere, observed in rocks, soils, and water resources. A potential threat to human health occurs when As enters the organism through the contaminated water supply or food. Since As engages in most biological catalytic reactions, its indirect or direct effect spreads to all organs [108]. The liver is the primary target for As poisoning [109]. The results of As toxicity are skin damage, cardiovascular and respiratory diseases, cancer, gangrene, brain dysfunction, etc. Meanwhile, there is currently no remedy endorsed for widespread use in As poisoning [110].

In turn, CUR has been shown to be a satisfactory antioxidant and protector for DNA, regressing As-induced damage in the nucleus (Table 3). Supplementation with CUR has been shown to reduce the total toxic load of As in the liver and assist to increase the As excretion through the urinary tract [111,112]. Moreover, CUR decreased transaminase and phosphatase activity along with the plasma and brain acetylcholinesterase, as well as total protein and albumin levels under As-induced liver injury [113].

Therefore, CUR may have some protective role against biochemical alterations and associated DNA damage caused by As. According to in vitro studies results, CUR modulates autophagy/apoptosis in cells and has a cytoprotective effect against As-induced toxicity, preventing decreased antioxidant levels that cause cell membrane disruption [114].

**Table 3:** Effects of curcumin on arsenic toxicity based on doses, experimental methods, and findings.

Dose/Concentration	Name of Animal Model/Cell Lines	Route of Exposure	Duration of Exposure/Treatment	Results	Source
**Clinical Trial**					
CUR + piperine (20:1) 2 × 500 mg/day	Chronically arsenic-exposed males or females	Orally	6 months	↓DNA damage, ↓ROS generation, ↓CAT, SOD enzymes,	[111]
CUR + piperine (100:1) 500 mg twice/day	Chronically arsenic-exposed males or females	Orally	6 months	↑expression of protein, mRNA of DNA-PK, DNA ligase IV, XRCC4, ↑BER and NHEJ repair pathways, ↓DNA-damaging effect in lymphocytes	[112]
**In Vivo**					
5 mg/kg b.w. NaAsO_2_ + 15 mg/kg b.w. CUR	Male Wistar rats	NaAsO_2_-orally/CUR-orally	30 days (co-administration)	↓transaminases, phosphatases, glucose, urea, creatinine, bilirubin, TL, cholesterol, TG, plasma and brain ache, the levels of TP and Alb	[113]
5 or 300 ppm NaAsO_2_ + 0.5 mg/kg b.w. nano-CUR	Male Swiss albino mice	NaAsO_2_-drinking water/CUR-orally	NaAsO_2_-7 days/CUR-14 days(post-treatment)	↓histopathological alterations, ↓accumulation of acidic vesicles, ↓apoptotic cells in the thymus and spleen, ↓autophagy, ↓redox imbalance in immune cells	[115]
**In Vitro**					
10 μM NaAsO_2_ + 0, 1, 2.5, 5, 10, 25, 50 or 100 μM CUR	PC12 cells	Cell line	24 h	↑membrane integrity, ↓DNA damage, apoptosis rate, ↑protein expressions, ↑cell viability, ↑cytoprotective effect, ↓oxidative stress	[114]

Abbreviations: ↑ = increase; ↓ = decrease; CUR = curcumin; As = arsenic; IP = intraperitoneal injection; TL = total lipids; TG = triglycerides; TP = total protein; Alb = albumin; ROS = reactive oxygen species; CAT = catalase; SOD = superoxide dismutase; DNA-PK = DNA-dependent protein kinase; XRCC = X ray repair cross complement; BER = base excision repair; NHEJ = nonhomologous end joining; NaAsO_2_ = sodium arsenite; PC = pheochromocytoma.

## 8. Curcumin on Cadmium-Induced Toxicity

Cadmium is a carcinogenic metal whose natural content gradually accumulates in the environment, due to anthropogenic emissions. The main route of Cd entry is skin absorption, ingestion, or inhalation. Cd with its wide range of toxic effects has become one of the most critical contaminants in the aquatic toxicology field owing to harmful human activity [116]. The problem that follows Cd accumulation is its competitive behavior with essential metals (Ca, Zn, Fe, Mg). Cd uses the same transport systems as essential metals, which blocks their entry into the cell, inducing toxicity [117,118].

Most studies have shown CUR’s positive healing properties against Cd toxicity (Table 4). Electrochemical studies in mice have predicted a compatible metal-ligand form of Cd and CUR, which could remove metal ions from the body [119] and mitigate the adverse effects of Cd [120]. It is reported that the oral administration of CUR decreased the accumulation of toxic elements in the brain and liver in mice [121]. Co-treatment with CUR significantly reduced the levels of pro-inflammatory cytokines such as tumor necrosis factor-alpha and interleukin-6, as well as biomarkers’ affection of oxidative damage [122]. CUR can inhibit the expression of nuclear factor kappa B, which is responsible for regulating the transcription of genes that control inflammation, immune cell development, and cell death. CUR prevented the release of interleukin-6 and interleukin-8 in response to toxic Cd ions [123]. The studies confirmed the CUR protective effects against Cd-induced nephrotoxicity. It also can be used to prevent respiratory tract injury due to Cd inhalation. Overall, CUR can be added to the diet and can be fairly proposed to be used as a protective factor for Cd-induced nephrotoxic, hepatotoxic, and hematological changes.

## 9. Curcumin on Copper-Induced Toxicity 

Copper’s redox nature makes it essential for many biological processes, but at the same time renders it toxic effects due to the generation of the most dangerous ROS, hydroxyl radical (^•^OH) [118]. An imbalance of Cu ions in the central nervous system engages in many neurodegenerative diseases pathogenesis, the most common is amyotrophic lateral sclerosis, Alzheimer’s, and Parkinson’s diseases [132,133,134,135]. For Cu toxicity, the same chelating treatment is used as for other metal poisonings [136].

Through quantum chemical computations, it was discovered that due to the proton loss of the CUR enol form, the β-diketone part is the main site of chelation in the CUR-Cu(II) complex [137] (Table 5). In studies conducted with CUR administration, CUR had ameliorating effects on the CuO nanoparticles toxicity in terms of the antioxidant, immunomodulatory, anti-apoptotic, and anti-inflammatory effects on the rats’ kidneys [138]. Moreover, considering the role of oxidative damage in mediating Cu toxicity, CUR suppressed neurotoxicity through its anti-radical and antioxidant properties [82]. CUR’s neuroprotective properties provide the basis for studying its effectiveness in several neurodegenerative diseases involving Cu, such as Alzheimer’s disease. Through the ability of CUR to penetrate the blood–brain barrier, CUR can block amyloid beta-peptide, a key disease factor that accumulates in the brain, and reduce its levels as well as remove the metal ions in the brain [139]. Thus, CUR as a restorative remedy for oxidative stress caused by Cu ions might be useful for therapeutic treatment, including neurodegenerative diseases.

## 10. Curcumin on Iron-Induced Toxicity

Iron is an indispensable element in basic life processes such as DNA synthesis, respiration, and a regular participant in biochemical reactions [143]. However, Fe overload in parenchymal organs is associated with degenerative changes in the cellular parenchyma that causes irreversible dysfunction of the most vulnerable vital organs, such as the liver, pancreas, and heart. Chelation therapy is the recommended approach as an antidote for Fe intoxication [144]. 

Some chelators are responsible both for Fe excretion and may suppress the participation of free labile Fe in oxidation-reduction reactions [94]. Age-related hypokinesia causes muscle atrophy which is related to the Fe accumulation and potentiates the oxidative stress through the Fenton reaction. Thus, the key role of chelating agent is to prevent the Fe participation in the Fenton reaction or the reaction of peroxides decomposition with the formation of hydroxyl radicals in the ROS generation [145].

CUR, an iron-binding antioxidant, prevents the destruction of cell membranes and stimulates the regeneration of hepatocytes, thereby having a protective effect on liver function in Fe overdose (Table 6). According to a study conducted on T51B cells treated with ferric ammonium citrate, CUR bounded but not blocked Fe absorption and did not interfere with its bioavailability [94]. CUR has shown a detoxifying effect by preventing the formation of ROS and abolishing the signaling cascades of Fe-induced stress response. Furthermore, in a study on MIN6 cells, CUR functioned as a moderator of Fe-dependent necrotic cell death, known as ferroptosis, and prevented Fe-induced cell damage during ROS production [146].

## 11. Curcumin on Lead-Induced Toxicity

Lead is a toxic metal whose compounds are insoluble in water but highly soluble in stomach acid. The main mechanism of its adverse action is associated with gastrointestinal tract disorders [149]. The Pb study’s importance is explained by its threatening toxicity through extreme spreading in the environment and its multilateral use in industry and everyday life. The toxic mechanism of Pb is associated with the blocking of thiol enzymes, interaction with biopolymers’ carboxyl and phosphate groups, nucleotides, and esterase enzyme inactivation [150,151].

Morphological and functional changes have been revealed that negatively affect the hematopoiesis and the nervous system in the fish body with oversaturated of Pb ions [152]. Fish are more sensitive to toxicity, the key role in the immune response in their body belongs to immunocompetent cells, primarily lymphocytes [153]. It has been reported that Pb led to a shift in white blood cell count and apoptosis has also been recorded [152]. A study conducted over 8 weeks with Pb added to aquarium water has shown an increase in fish mortality and stunting along with a decrease in indicators such as final wet weight and specific growth rate. However, after co-treatment with CUR, DNA damage was markedly reduced, the effects of Pb toxicity in the kidneys were weakened, and the inflammatory process was attenuated [154] (Table 7).

Pb increases lipid peroxidation and reduces the functional abilities of the brain cell, but CUR significantly reduced the Pb toxic effect on nerve cells in rat studies [155]. Furthermore, in rat experiments with Pb accumulation in the liver and kidneys, it was shown that CUR is an effective inducer of the post-accumulation effects of oxidative toxicity. The results have shown a decrease in the Pb amount in the affected organs and the relief of degenerative changes caused by Pb [156].

**Table 7:** Effects of curcumin on lead toxicity based on doses, experimental methods, and findings.

Dose/Concentration	Name of Animal Model/Cell Lines	Route of Exposure	Duration of Exposure/Treatment	Results	Source
**In Vivo**					
50 mg/kg Pb(C_2_H_3_O_2_)_2_ + 100 mg/kg CUR 50 mg/kg Pb(C_2_H_3_O_2_)_2_ + 200 mg/kg CUR	Male Sprague–Dawley rats	Pb(C_2_H_3_O_2_)_2_, CUR- orogastric tube	Pb(C_2_H_3_O_2_)_2_-4 weeks/CUR-4 weeks (post-treatment)	↓Pb concentration, ↓oxidative stress, ↓cerebellar damage in cerebellum, ↑motor coordination	[155]
50 mg/kg Pb(C_2_H_3_O_2_)_2_ + 200 mg/kg CUR	Male Wistar rats	Pb(C_2_H_3_O_2_)_2_ -IP/CUR-orally	7 days (co-administration)	↓apoptosis, ↓oxidative stress, ↓inflammation, ↓liver injury, ↑AKT/GSK-3β signaling pathway	[157]
20 mg/kg OD Pb(C_2_H_3_O_2_)_2_ + 30 mg/kg BD CUR	Male and female Wistar rats	Pb(C_2_H_3_O_2_)_2_, CUR- IP	5 days (co-administration)	↓damage neurons, ↓protein oxidation, lipid peroxidation, ↑GSH	[158]
1 mg/L Pb + 15 g/kg CUR	*Cyprinus carpio*	Pb-aquarium water/CUR-orally	8 weeks (co-administration)	↓mRNA, expression of NF-kB p65, ↓AST, ALT, LDH, ↑protease activity, ↑SOD, GPx, GSH, G-Rd, GST, Nrf2, ↑lysozyme activity, C3, IgM, ↑intestinal microbial abundance, ↑growth parameters, ↑RBC, Hct, Hb, serum protein, albumin, ↑enzymatic activities, ↑IL-10, ↓MDA	[154]
**In Vitro**					
10 μM Pb(C_2_H_3_O_2_)_2_ + 50, 100, or 150 μM CUR	Rat pups’ hippocampi		3 h	↓free radicals, ↑cell viability	[158]

Abbreviations: ↑ = increase; ↓ = decrease; CUR = curcumin; Pb = lead; IP = intraperitoneal injection; AKT = protein kinase B; GSK-3β = glycogen synthase kinase-3β; OD = once daily; BD = twice daily; SOD = superoxide dismutase; GPx = glutathione peroxidase; GSH = glutathione; IL-10 = interleukin-10; MDA = malondialdehyde; GST = glutathione S-transferase; G-Rd = glutathione reductase; LDH = lactate dehydrogenase; RBC = red blood cells; Hct = hematocrit; Hb = hemoglobin; NF-κB p65 = nuclear factor kappa B p65 subunit; AST = aspartate transaminase; ALT = alanine transaminase; Nrf2 = nuclear factor erythroid 2–related factor 2; C3 = complement component 3; Pb(C_2_H_3_O_2_)_2_ = lead acetate.

## 12. Curcumin on Zinc-Induced Toxicity

Zinc is an essential trace element for all life forms. Zn is part of more than 40 enzymes and engages in carbohydrate metabolism and the metabolism of vitamin A. This element is necessary for bone formation. In addition, it exhibits antiviral and antitoxic effects, and Zn toxicity has rarely been reported [159]. 

Zinc oxide nanoparticles (ZnONP) are of major interest, as they are widely distributed in various fields. ZnONP are used in pharmaceuticals and cosmetology as agents exhibiting antibacterial activity. However, along with beneficial properties, the harmful effect of ZnONP on various body systems has been recorded [160]. Therefore, the toxic effects of Zn and ZnONP require a protective agent. CUR has shown promising results in preventing Zn toxicity in vivo studies (Table 8). Analyzing the content of ZnONP in the brain after administration on rats, it has been found that nanoparticles or its metabolic products cross the blood–brain barrier and cause histological damage to the cerebellum, which in turn was successfully prevented after the addition of CUR [52]. 

CUR is also proven to be a successful Zn ions chelator and can function as a radical scavenger that accumulates Zn ions [161]. Therefore, apoptosis and necrosis of hepatocytes can be prevented by suppressing the oxidative damage of cells through the CUR treatment [162]. In addition, CUR is a vindicated protector of nephrotoxicity which can reduce kidney damage, and suppresses necrosis factors, and inflammation processes [163].

**Table 8:** Effects of curcumin on zinc toxicity based on doses, experimental methods, and findings.

Dose/Concentration	Name of Animal Model/Cell Lines	Route of Exposure	Duration of Exposure/Treatment	Results	Source
**In Vivo**					
5.6 mg/kg b.w. ZnONP + 200 mg/kg CUR	Male albino rats	ZnONP-IP/CUR-oral gavage	ZnONP-28 days (started on day 7, three times per week)/CUR-28 days (pre-treatment)	↑cerebellum structure, ↓oxidative stress markers, ↑inflammatory response, ↓COX-2, P53	[52]
50 mg/kg ZnONP + 200 mg/kg CUR	Male Wistar rats	ZnONP, CUR-orally	ZnONP-14 days (started on day 7)/CUR-14 days (pre-treatment)	↑body, kidney weight, ↓MDA in the renal tissue, ↑SOD, GPx, ↓histological changes, ↓apoptotic index	[162]
50 mg/kg nano-ZnO + 200 mg/kg CUR	Male Wistar rats	ZnO-gavage method/ CUR- oral gavage	ZnO-21 days (started on day 7)/CUR-21 days (pre-treatment)2	↓lipid peroxidation, ↑SOD, GPx ↓ALT, AST, ALP, ↓histology changes, ↓apoptotic index of hepatocytes	[163]

Abbreviations: ↑ = increase; ↓ = decrease; CUR = curcumin; Zn = zinc; ZnO = zinc oxide; ZnONP = zinc oxide nanoparticles; IP = intraperitoneal injection; P53 = tumor protein P53; COX-2 = prostaglandin-endoperoxide synthase 2; MDA = malondialdehyde; SOD = superoxide dismutase; GPx = glutathione peroxidase; ALT = alanine aminotransferase; AST = aspartate aminotransferase; ALP = alkaline phosphatase.

## 13. Curcumin on Mercury-Induced Toxicity

Mercury in its natural state at room temperature is a liquid metal. Humans typically contact with Hg from three main sources: dental amalgam fillings, some vaccines, and fish caught in rivers and seas contaminated with Hg waste [10,164]. In addition, Hg-containing devices—such as electric lamps, thermometers, barometers, etc.—are potential sources of Hg in the home [165,166]. 

Chronic Hg poisoning can lead to ataxia, a lack of coordination that causes a cerebellar gait. It can also cause hand tremors, excessive salivation, and a metallic taste in the mouth. Another representative symptom could be a blue line that appears on the alveolar margin of the gums, as in Pb and bismuth poisoning. The classic symptoms of Hg vapor poisoning include intention tremors, erethism (memory loss, lack of self-control, irritability, excitability, loss of self-confidence, drowsiness, and depression), and gingivitis [167].

CUR has been examined in several Hg-induced toxicity studies (Table 9). CUR treatment provided protective effects on Hg-induced oxidative stress parameters, and it was found to be effective in reversing Hg-induced serum biochemical changes [168]. However, it also reported that CUR supplementation in excess of metal ions from Hg, Al, or As can inhibit utilization of some essential trace minerals such as Se or Zn which may ultimately affect the antioxidant defense system. Similarly, it showed that CUR as a strong antioxidant can limit the levels of other antioxidants in the liver to maintain the optimum antioxidant levels in organisms [168]. 

Pi3K-Akt has been proposed as a key signaling pathway in Hg-induced splenic injury. In vivo experiment in mice has shown that CUR attenuated spleen apoptosis by reversing the same pathway as well as mitigated HgCl_2_-induced toxicity in the immune system through the Nrf2-ARE activation pathway [169]. Here, the protective role of Nrf2, once released from Keap1, is mainly derived from the activation at the level of the antioxidant response elements (ARE), thereby giving rise to the production of antioxidant enzymes. In another study, it has examined the effects of Hg on neurobehavioral and neurochemical disorders in mouse offspring where CUR increased the levels of cerebral monoamines (dopamine, norepinephrine, and serotonin) in Hg-treated pups. Overall, behavioral disorders, such as anxiety behavior, were suppressed by CUR in treating pups. Conclusively, CUR has shown a desirable mechanism in preventing Hg toxicity and could be recommended for use to avoid toxicants such as Hg [170].

## 14. Curcumin on Selenium-Induced Toxicity

Selenium is an indispensable, vital trace element for the organisms [151]. It is represented by the active center of many Se-containing proteins involved in the antioxidant defense mechanisms, thyroid hormone metabolism, and performing the immune function [171].

Se supports the Se-containing enzymes function and selenoproteins contained in plasma [10]. Se affects the metabolism of leukotriene, thromboxane, and prostacyclin. Se deficiency disrupts the functioning of the brain [172] and suppresses immune defense reactions, especially nonspecific, cellular, and humoral immunity [173].

In excess concentration, Se can exhibit toxic properties [174]. Symptoms of toxic effects of excess Se include a metallic taste, headaches, nausea, hair loss, and damaged nails. Sensory loss, convulsions, pneumonia, pulmonary edema, and circulatory collapse are also symptoms. Cases of Se toxic effects have been registered not only during exposure to Se associated with industrial production but also during its self-administration [174].

Studies of the role of CUR in combating Se-induced toxicity in the liver and kidneys of Wistar rats have yielded promising results (Table 10). Kidney tissue of Se alone administered rats was critically damaged: cystic degeneration, cytoplasmic vacuolization, cellular proliferation with fibrosis, and vesicle formation. Conversely, CUR pretreatment prevented all degenerative changes caused by Se [175]. There have been several studies of Se-induced cataractogenesis with CUR co-treatment. The findings showed that CUR suppressed Se-induced accumulation of reactive oxygen species and cataract formation in the isolated lens from experimental rat pups, possibly due to inhibition of the enzymatic and non-enzymatic antioxidant defense system and prevention of uncontrolled formation of superoxide radicals, as well as due to inhibition of iNOS activity [176,177].

The results implied that CUR might take place as an agent against hepatic and renal toxicities, probably due to its ability to inhibit iNOS levels and proved an antioxidant potential of CUR in preventing Se-exposed toxicity.

## 15. Curcumin on Chromium-Induced Toxicity

Chromium compounds are highly toxic to humans, primarily hexavalent chromium (Cr^+6^). The main manifestations of excess Cr are inflammatory diseases with a tendency to ulceration of the mucous membranes, an allergic effect, dermatitis and eczema, bronchial asthma, and risk of cancer [178,179].

The studies reported the effects of CUR against Cr toxicity in the male reproductive system has detected success in the prevention of the Cr destructive action in studied parameters (body weight, the weight of the testis, accessory sex organ weight) (Table 11). CUR through its antioxidant properties protected male germ cells, and testicular histology from oxidative damage by Cr-induced free radicals [180]. The study on Cr-induced renal dysfunction has reported that CUR treatment attenuated tissue damage, decreased free radicals, and reduced antioxidant factors in both kidney tissue and mitochondria. Moreover, the authors highlighted that the protection of mitochondrial function played a crucial role in the defense mechanism of CUR pretreatment against Cr toxicity in the kidneys [181]. Thus, the present studies suggest that CUR may have a protective role against Cr toxicity.

## 16. Conclusions and Future Outlook

The presented literature data provide a wide range of biological activity that determines the various therapeutic properties of curcumin, the polyphenolic compound of the *Curcuma longa* plant, in relation to metal toxicity. The therapeutic and prophylactic efficacy of curcumin has been found in many inflammatory, oncological, autoimmune, and neurodegenerative diseases. Drugs for the treatment of these diseases are often expensive and have certain side effects. In this regard, the importance of using multi-purpose, harmless, inexpensive, and readily available dietary supplements or nutraceuticals for the prevention and treatment of various human diseases is increasing [60]. 

Various modern forms (capsules, tablets, lozenges) of dietary supplements containing turmeric, and its bioactive compounds are commercially available. Turmeric powder with glycerin has been used in the treatment of a burning sensation in oral submucous fibrosis [182]. An ointment containing 10% alcohol extract from turmeric roots is effective in the treatment of ulcers caused by aspirin [183]. Turmeric is a safe and secure alternative remedy in the treatment of digestive disorders [184]. It is recommended as a means of stimulating gastric secretion and normalizing the intestines and lungs functioning [185,186,187,188]. Turmeric oil has anti-ulcerogenic potential and prevents the formation of stomach ulcers [189,190,191]. The anti-*Helicobacter pylori* therapeutic effect was increased with the curcumin supplement therapy [192,193]. Satisfactory results have been obtained using curcumin as a supplement in the treatment of ulcerative colitis, and Crohn’s disease [194]. A mixture of turmeric and ginger oils has a strong hepatoprotective effect in acute alcohol intoxication [195]. Curcumin prevents the development of non-alcoholic fatty liver induced by a high-cholesterol diet [196]. Turmeric is effective in gallbladder diseases [197]. Dietary supplements with curcumin have high bioavailability at recommended dose, good tolerance, and minimal signs of toxicity, which can only appear in individuals with intolerance [54,58]. 

The low toxicity, even at doses up to 8 g per day [60], availability, and high activity of turmeric and curcumin make it possible to use them for a long time both with food in the form of spice and as a purified biologically active component, which is promising for the prevention of the above diseases and use as an adjuvant in their complex therapy [63,198]. However, this polyphenol has not been approved for clinical use yet.

Numerous studies confirm the effectiveness of curcumin in various pathological processes, however, its pure use is limited by its low bioavailability [199]. Therefore, one of the important technological tasks is the development of effective dosage of curcumin to increase the bioavailability of this compound.

To improve the bioavailability of curcumin, scientists propose various methods. These include the use of enhancers, compounds that promote the delivery of biologically active substances, such as the alkaloid piperine, which prevents glucuronidation; incorporation of curcumin into liposomes and nanoparticles; use of the curcumin-phospholipid complex; complexes with cyclodextrins; etc. By increasing oral bioavailability, curcumin can be proposed as a new orally active metal chelator, such as deferasirox, for the treatment of metal toxicity. 

Curcumin is a suitable candidate for metal toxicity treatment given its wide range of biological effects [200]. Detoxification processes triggered by the protective effects of curcumin have been linked to its ability to scavenge free radicals, function as a natural chelating agent, and/or induce antioxidant enzymes by initiating the Keap1/Nrf2/ARE pathway. Even though the molecular mechanisms and primary targets of this compound are still unclear [201]. These circumstances put a barrier to the recognition of curcumin as a potential pharmacological agent, but do not prevent its use as a biologically active agent. Thus, curcumin with an improved form of bioavailability may be used as an adjuvant in the treatment of metal poisoning. The chemical specificity of curcumin may be a useful factor in discovering unknown therapeutic targets in metal toxicology, but more research is needed in this area.

## Figures and Tables

**Table 1:** Average tolerable intake of metals for a person.

Metal	HBGV	Value	Source
Aluminum	TWI	2000 μg/kg b.w.	[12]
	TDI	10,000 μg/kg b.w.	[13]
Arsenic	TWI	15 μg/kg b.w.	[14]
Cadmium	TWI	2.5 µg/kg b.w.	[15]
Chromium	TDI	300 μg/kg b.w.	[16]
Cobalt	TDI	1.4 μg/kg b.w.	[17]
Copper	UL	10,000 μg/kg b.w.	[18]
Iron	TDI	800 µg/kg b.w.	[19]
Lead	TWI	25 µg/kg b.w.	[20]
	TDI	600 µg/kg b.w.	
Manganese	TDI	60 µg/kg b.w.	[21]
Mercury	TWI	4 µg/kg b.w.	[22]
Nickel	TDI	12 μg/kg b.w.	[23]
Selenium	UL	400 μg/kg b.w.	[24]
Zinc	UL	40,000 μg/day	[18]

Footnote: The intake of metals depends on the concentration of metals in food and daily food intake. In addition, tolerance to contaminants can be influenced by body weight [11]. A Tolerable Daily or Weekly Intake (TDI/TWI) is the maximum harmless daily dose of a chemical pollutant for a person that does not cause any adverse effects during the daily or weekly intake throughout life. Abbreviations: b.w. = body weight; HGBV = health-based guidance values; UL = tolerable upper intake level; TWI = tolerable weekly intake; TDI = tolerable daily intake.

**Table 2:** Effects of curcumin on aluminum toxicity based on doses, experimental methods, and findings.

Dose/Concentration	Name of Animal Model/Cell Lines	Route of Exposure	Duration of Exposure/Treatment	Results	Source
100 mg/kg b.w. Al + 50 mg/kg b.w. CUR	Sprague–Dawley rats	Al-drinking water/CUR-IP	Al-8 weeks/CUR-2 months (co-administration)	↓TNF-α, ↓NF-kB p65, ↓NO activity	[100]
50 mg/kg/day Al + 30 mg/mL/kg b.w. CUR	Male Wistar rats	Al-drinking water/CUR-orally	6 months (co-administration)	↓lipid peroxidation in the brain, ↓SOD, GPx, GST and Na+, K+, ATPase	[107]
100 mg/kg Al + 30 or 60 mg/kg CUR	Male Wistar rats	Al-drinking water/CUR-orally	42 days(co-administration)	↓IAL, first and second RL to reach the platform in the pre-trained rats, ↑retention performance of the spatial navigation task; ↓MDA, nitrite levels, ↑reduced GSH, ↓GST, SOD, and catalase activity, ↓AChE activity, ↓Al in hippocampus	[104]
	Synthesized [Al(CUR) (EtOH)_2_](NO_3_)_2_) complex	Binding of CUR complex to calf thymus-DNA		↓affinity of Al to interact with DNA	[103]
	Synthesized Al (III)–CUR complexes	NMR, mass spectroscopy, ultraviolet, the generalized 2D UV–UV correlation spectroscopy, the density functional theory		↓affinity of Al to interact with amyloid beta (Aβ) peptide, ↓ toxicity effect of Al on peptides, ↓ oxidative stress	[102]

Abbreviations: ↑ = increase; ↓ = decrease; CUR: curcumin; Al = aluminum; IP = intraperitoneal injection; TNF-α = tumor necrosis factor alpha; NF-κB p65 = nuclear factor kappa B p65 subunit; NO = nitric oxide; SOD = superoxide dismutase; GPx = glutathione peroxidase; GST = glutathione S-transferase; ATPase = adenosine triphosphatase; IAL = initial acquisition latencies; first RL = first retention latency; second RL = second retention latency; AChE = acetylcholinesterase; NMR = Nuclear magnetic resonance.

**Table 4:** Effects of curcumin on cadmium toxicity based on doses, experimental methods, and findings.

Dose/Concentration	Name of Animal Model/Cell Lines	Route of Exposure	Duration of Exposure/Treatment	Results	Source
**In Vivo**					
0.8 mg/L water Cd + basal diet with 0.5% TP	Juvenile African catfish	Cd-aquarium water/TP-orally	30 days (co-administration)	↑growth rate, ↑HSI, ↓damage to the hepatic architecture, the testis architecture, ↑reproduction hormone level, lysozyme activity, immunoglobulin levels, protein content, ATP content, ↓disturbed enzyme activities, ↓nephrotoxicity, ↓oxidative stress, ↓apoptotic events, ↓Cd accumulation in muscles, the liver	[124]
40 mg/L CdCl_2_ + 50 mg/kg b.w. CUR	Male albino rats	Cd-drinking water/CUR-gastric tube	6 weeks (co-administration)	↓TNF-α, IL-6, ↓lipid peroxidation, ↑CAT, SOD, GSH, TAC, ↓oxidative stress, ↓loss of antioxidant enzymes	[122]
200 mg/kg b.w. C_4_H_6_CdO_4_ + 250 mg/kg b.w. CUR	Adult male rats	Cd-drinking water/CUR-orally	7 days (co-administration)	↓level of urea, creatinine in the serum, ↑antioxidant levels	[125]
1 mg/kg b.w. Cd + 100 mg/kg b.w. CUR	Male Wistar albino rats	Cd-subcutaneously/CUR-intragastric intubation	4 weeks (co-administration)	↓testicular damage, ↓reactivity and the number of germ cell, Leydig cell apoptosis, ↓TUNEL positive cells of testis	[126]
1 mg/kg/day or 100 mg/kg/day CdCl_2_ + 1 mg/kg/day or 100 mg/kg/day CUR	Male Spraque–Dawley rats	CdCl_2_-IP/CUR-orally	3 days (co-administration)	↓TBARS levels, ↑GSH, CAT, GPx, SOD, ↓histopathological changes, ↓lipid peroxidation, ↓oxidative stress in testis tissue, ↑antioxidant enzymes	[127]
7 mg/kg b.w. CdCl_2_ + 50 mg/kg b.w. CUR	Male CD mice	CdCl_2_- subcutaneously/CUR-orally	CdCl_2_-ones/CUR-3 days (pre-treatment)	↑GSH, GPx, ↑antioxidant status, ↔Cd level in tissues, ↓lipid peroxidation	[121]
0.025 mmol/kg b.w. (rats), 0.03 mmol/kg b.w. (mice) CdCl_2_ + 50 mg/kg b.w. CUR	Male Wistar rats, male CD mice	CdCl_2_- subcutaneously/CUR-orally	CdCl_2_-ones/CUR-3 days (pre-treatment)	↓lipid peroxidation	[128]
5 mg/kg b.w. CdCl_2_ + 200 or 400 mg/kg b.w. CUR	Adult male Wistar rats	CdCl_2_- oral gavage/CUR-oral gavage	27 days (co-administration)	↓MDA, ↑GSH, ↓nephrotoxicity	[129]
5 mg/kg b.w. CdCl_2_ + 200 or 400 mg/kg b.w. CUR	Adult male ICR mice	Cd-drinking water/CUR-intragastric	8 weeks (co-administration)	↓systolic, diastolic, and mean arterial blood pressure levels, ↑Phe, Ach, and SNP, ↓hypertension, ↓impairment of vascular responsiveness to vasoactive agents, ↓lipid peroxidation, ↓protein oxidation, ↑GSH, ↑redox status of the blood cells, ↓oxidants, ↑endogenous antioxidant formation, ↓oxidative stress, ↓Cd accumulation in the blood, organs	[130]
**In Vitro**					
100 mM CdSO_4_ + 10, 30, 20, 40 or 50 μM CUR	Bronchial epithelial cell line Calu-3		24 h	↓IL-6, IL-8 mRNA transcript levels, ↓Erk1/2 activation	
0.025, 0.05, 0.1 mM CdCl_2_ + 0.025, 0.05, 0.1 mM CUR	Rat brain homogenate	Lipid peroxidation assay		↓lipid peroxidation	[131]

Abbreviations: ↑ = increase; ↓ = decrease; ↔ = no change; CUR = curcumin; Cd = cadmium; TP = turmeric powder; ATP = adenosine triphosphate; HIS = hepatosomatic index; CAT = catalase; SOD = superoxide dismutase; TNF-α = tumor necrosis factor-alpha; IL-6 = interleukin-6; IL-8 = interleukin-8; GSH = glutathione; TAC = total antioxidant capacity; Erk1/2 = extracellular signal-regulated protein kinase 1/2; TUNEL = terminal dUTP nick end-labeling assay; GPx = glutathione peroxidase; TBARS = thiobarbituric acid reactive substance; MDA = malondialdehyde; SNP = sodium nitroprusside; Ach = acetylcholine; Phe = phenylephrine; CdCl_2_ = cadmium chloride.

**Table 5:** Effects of curcumin on copper toxicity based on doses, experimental methods, and findings.

Dose/Concentration	Name of Animal Model/Cell Lines	Route of Exposure	Duration of Exposure/Treatment	Results	Source
**In Vivo**					
100 mg/kg CuSO_4_ + 80 mg/kg CUR; 100 mg/kg CuSO_4_ + 80 mg/kg nano-CUR	Male Wistar rats	Cu-orally/CUR-orally	7 days (co-administration)	↓cerebral oxidative stress, ↓cerebral inflammation in stress, ↓apoptosis, ↑AKT/GSK-3β signaling pathway, ↑BDNF	[82]
250 mg/kg CuONP + 200 mg/kg b.w. CUR	Rats	CuONP-oral gavage/CUR- orally	3 months (co-administration)	↑body weight gain, ↓serum creatinine, BUN levels, ↓KIM-1 in urine, kidney, ↓oxidative stress, ↓NO level, ↓mRNA expression of IL1-β, TNF-α, NF-ĸB ↑Nrf2, HO-1, γ-GCS gene expression in the kidney, ↓renal damage, ↓morphological tubular, glomerular alteration, ↓caspase-3	[138]
1 mM Cu + 0.2 or 0.5 mg/kg CUR	*Drosophila melanogaster* wild-type (Harwich strain) flies	Cu, CUR-medium	7 days (co-administration)	↓oxidative stress, ↓nitrite level, ↓AChE activity, ↓dopamine levels	[140]
4 mg/kg CuSO_4_ + 80 mg/kg CUR	Albino rats	CuSO_4_, CUR- orally by stomach tube	30 days (co-administration) CuSO_4_-15 days/CUR-15 days (post-treatment)	↓hepatic marker enzymes, ↑hepatic, renal antioxidants, MDA, ↓renal tissue damage, ↑antioxidant enzymes, ↓ROS	[141]
**Other**					
Cu^2+^, Zn^2+^, Fe^2+^ + CUR		Spectrophotometry		Binding more readily Fe and Cu than Zn, ↓Aβ toxicity, ↓NF-κB	[142]

Abbreviations: ↑ = increase; ↓ = decrease; CUR: curcumin; Cu = copper; BDNF = brain-derived neurotrophic factor; AKT = protein kinase B; GSK-3β = glycogen synthase kinase-3β; CuONP = copper (II) oxide nanoparticles; BUN = blood urea nitrogen; KIM-1 = kidney injury molecule-1; NO = nitric oxide; IL1-β = interleukin-1β; TNF-α = tumor necrosis factor-alpha; NF-ĸB = nuclear factor-κB; Nrf2 = nuclear factor-erythroid factor 2-related factor 2; HO-1 = heme oxygenase-1; γ-GCS = γ-glutamylcysteine synthetase; AChE = acetylcholinesterase; MDA = malondialdehyde; ROS = reactive oxygen species; CuSO_4_ = copper sulfate.

**Table 6:** Effects of curcumin on iron toxicity based on doses, experimental methods, and findings.

Dose/Concentration	Name of Animal Model/Cell Lines	Route of Exposure	Duration of Exposure/Treatment	Results	Source
**In Vivo**					
30 mg FeSO_4_ in 1 mL saline ⁄ kg b.w. + basal diet with 0.2 g% CUR	Male Wistar rats	FeSO_4-_IP/CUR- oral gavage	FeSO_4_-ones/CUR-8 weeks (pre-treatment)	↓LDL oxidation, ↓ALT, AST, LDH, ↓liver lipid peroxide level, ↓severity of hepatotoxicity	[147]
**In Vitro**					
250 µM or 500 µM Fe-NTA + 20 μM or 50 μM CUR	Huh-7, T51B, RL-34 cells		24, 48, 72, 96, 120 h	↑tumor-promoting effect, ↑Fe^2+^ chelating, ↔Fe uptake	[94]
200 µM FAC + 50 µM CUR	T51B cell line		5 or 10 days	↑Fe chelating, ↔Fe uptake/bioavailability, ↓ROS, ↓signaling to cellular stress pathways,	[148]
50 μmol/L FAC or 30 μmol/L FeSO_4_ + 20 μM CUR	Mouse MIN6 pancreatic β-cell line		24 h	↓cell damage, ↑stress protection, ↓cell mortality, ↓Fe accumulation, ↓MDA, ↑GSH	[146]

Abbreviations: ↑ = increase; ↓ = decrease; ↔ = no change; CUR = curcumin; Fe = iron; IP = intraperitoneal injection; ALT = alanine aminotransferase; AST = aspartate aminotransferase; LDH = lactic dehydrogenase; ROS = reactive oxygen species; Fe-NTA = ferric nitrilotriacetate; FAC = ferric ammonium citrate; MDA = malondialdehyde; GSH = glutathione; FeSO_4_ = iron (II) sulfate.

**Table 9:** Effects of curcumin on mercury toxicity based on doses, experimental methods, and findings.

Dose/Concentration	Name of Animal Model/Cell Lines	Route of Exposure	Duration of Exposure/Treatment	Results	Source
**In Vivo**					
12 µmol/kg b.w. HgCl_2_ + 80 mg/kg b.w. CUR	Male Wistar rats	HgCl_2_-IP/CUR-orally	HgCl_2_-ones(pre/post-treated groups)/CUR-3 days(post-treatment, pre-treatment)	↓oxidative stress, ↓lipid peroxidation, ↑GSH, SOD GPx, CAT in the liver, kidney, brain, ↓serum biochemical changes, ↓Hg in tissues	[168]
5 mg/kg HgCl_2_ + 50 mg/kg b.w. CUR	Kunming male mice	HgCl_2_, CUR-IP	HgCl_2_-ones/CUR-24 h (pre-treatment)	↓autophagic cell death, ↓Na overload, ↓Ca leak, ↑Nrf2 signaling pathway, ↑antioxidant defenses	[169]
10 ppm HgCl_2_ + 150 or 300 ppm CUR	Pregnant Swiss-Webster strain mice	HgCl_2_, CUR-oral gavage	15 days (co-administration)	↓biochemical, behavioral disorders, ↑cognitive, anxiety behaviors	[170]

Abbreviations: ↑ = increase; ↓ = decrease; CUR = curcumin; Hg = mercury; IP = intraperitoneal injection; SOD = superoxide dismutase; GPx = glutathione peroxidase; GSH = glutathione; Nrf2 = nuclear factor erythroid 2–related factor 2; Ca = calcium; Na = sodium; HgCl_2_ = mercury (II) chloride.

**Table 10:** Effects of curcumin on selenium toxicity based on doses, experimental methods, and findings.

Dose/Concentration	Name of Animal Model/Cell Lines	Route of Exposure	Duration of Exposure/Treatment	Results	Source
**In Vivo**					
15 µM/kg b.w. Se + 75 mg/kg b.w. CUR	Wistar rats	Se-IP/CUR-orally	Se-ones(co/pre/post-treated groups)/CUR-ones (co-administration, post-treatment, pre-treatment)	↓liver cells damage, ↓architecture changes of glomeruli and tubules, ↓iNOS	[175]
15 µM/kg b.w. Se + 75 mg/kg b.w. CUR	Wistar rat pups	Se-IP/CUR-orally	Se-ones(co/pre/post-treated groups)/CUR-ones (co-administration, post-treatment, pre-treatment)	↓LPO, ↓SOD, GST, GPx, CAT, ↓GSH, vitamin C, vitamin E	[177]
**In Vitro**					
100 μM Na_2_SeO_3_ + 100 μM or 200 μM CUR	Wistar Rat pups	Isolated lenses incubation in DMEM	62 h	↓oxidative stress, ↓cataract formation	[176]

Abbreviations: ↑ = increase; ↓ = decrease; CUR = curcumin; Se = selenium; IP = intraperitoneal injection; iNOS = inducible nitric oxide synthase; DMEM = Dulbecco’s modified eagle medium; SOD = superoxide dismutase; GPx = glutathione peroxidase; GST = glutathione S-transferases; GSH = glutathione; CAT = catalase; LPO = lipid peroxidation; Na_2_SeO_3_ = sodium selenite.

**Table 11:** Effects of curcumin on chromium toxicity based on doses, experimental methods, and findings.

Dose/Concentration	Name of Animal Model/Cell Lines	Route of Exposure	Duration of Exposure/Treatment	Results	Source
**In Vivo**					
0.4 mg/kg b.w. OD K_2_Cr_2_O_7_ + 20 mg/kg b.w. CUR	Male albino rats of Sprague–Dawley strain *Rattus norvegicus*	K_2_Cr_2_O_7_,CUR-IP	K_2_Cr_2_O_7_-26 days/CUR-26 days(every alternate day) (co-administration)	↓testicular histology damage, ↑sperm count, ↑testosterone level, ↑accessory sex organs weight, ↓lipid peroxidation, ↑SOD, CAT	[180]
015 mg/kg b.w. K_2_Cr_2_O_7_ + 100, 200 or 400 mg/kg b.w. CUR	Male Wistar rats	K_2_Cr_2_O_7_- subcutaneously/CUR- gavage	K_2_Cr_2_O_7_-ones(co/pre/post-treated groups)/ /CUR- 10 days before and 2 days after K_2_Cr_2_O_7_ injection (co-administration, post-treatment, pre-treatment)	↓renal dysfunction, ↓histological damage, ↓oxidant stress, ↑antioxidant enzymes, ↔Cr in renal homogenates, in mitochondria (pre-treated), ↑mitochondrial oxygen consumption, ↑activity of mitochondrial complexes I, II, II–III, V, ↑mitochondrial ATP content, ↑mitochondrial Ca^2+^ transport, mitochondrial membrane potential, ↔Cr concentration, ↑nuclear translocation of Nrf2, ↑GST	[181]

Abbreviations: ↑ = increase; ↓ = decrease; ↔ = no change; CUR = curcumin; Cr = chromium; IP = intraperitoneal injection; SOD = superoxide dismutase; CAT = catalase; GST = glutathione S-transferases; Nrf2 = Nuclear factor-erythroid factor 2-related factor 2; K_2_Cr_2_O_7_ = potassium dichromate.

## Data Availability

All data is reported in this article.
